# WISDOM Randomized Trial Comparing Risk-Based Versus Annual Breast Cancer Screening: Study Cohort Characteristics and Design

**DOI:** 10.21203/rs.3.rs-7665355/v1

**Published:** 2025-10-05

**Authors:** Allison Fiscalini, Kelly Blum, Kirkpatrick Fergus, Robert A. Hiatt, Arash Naeim, Andrea Kaster, Maren T Scheuner, Laura J van ‘t Veer, Andrea Z LaCroix, Alexander D Borowsky, Olufunmilayo I. Olopade, Hannah L Park, James Esserman, Rachael Lancaster, Diane Heditsian, Susie Brain, Vivian Lee, Deborah L Goodman, Katherine S Ross, Neil Wenger, Barbara A Parker, Lisa Madlensky, Jeffrey A Tice, Yiwey Shieh, Elad Ziv, Vignesh Arasu, Hoda Anton-Culver, Celia P. Kaplan, Kim F Rhoads, Kimberly Badal, Michael Hogarth, Antonia Petruse, Irene A. Soto, Katherine Leggat-Barr, Steffanie Goodman, Jennifer Atamer, Rita Ryu, Jeffrey B Matthews, Kelly Adduci, Martin Eklund, Leah P Sabacan, Laura J Esserman

**Affiliations:** University of California San Francisco; University of California San Francisco; University of California San Francisco; University of California San Francisco; University of California Los Angeles; Sanford Health; San Francisco VA Health Care System; University of California San Francisco; University of California San Diego; University of California San Francisco; University of Chicago; University of California Irvine; Diagnostic Center of Miami; University of Alabama at Birmingham; University of California San Francisco; University of California San Francisco; University of California San Francisco; University of California Irvine; University of California San Francisco; University of California Los Angeles; University of California San Diego; University of California San Diego; University of California San Francisco; Weill Cornell Medicine; University of California San Francisco; Kaiser Permanente; University of California Irvine; University of California San Francisco; University of California San Francisco; University of California San Francisco; University of California San Diego; University of California Los Angeles; University of California San Francisco; Tufts University; University of California San Francisco; University of California San Francisco; University of California San Diego; University of California San Francisco; University of California San Francisco; Karolinska Institutet; University of California San Francisco; University of California San Francisco

**Keywords:** Randomized Controlled Trial, Breast Cancer, Screening, Prevention, Polygenic Risk Score

## Abstract

The Women Informed to Screen Depending on Measures of risk (WISDOM) Study (NCT02620852) is pragmatic randomized controlled trial evaluating risk assessment to inform the age to start, frequency and modality of breast cancer screening and risk reduction. The WISDOM Study uses a preference-tolerant design, enabling women who decline randomization to self-select their screening arm. The risk-based arm included comprehensive breast cancer risk assessment using the Breast Cancer Surveillance Consortium (BCSC) model and genetics. From August 2016 – February 2023, WISDOM enrolled a large, diverse, nationwide cohort of 46,289 participants – 77% non-Hispanic (NH) White, 9% Hispanic, 6% (NH) Black, and 5% (NH) Asian; demographic diversity increased over time. Mean age at baseline was 54 years. 28,335 (61.2%) were randomized, while 89% of those who self-selected their screening arm chose risk-based screening. The preference-tolerant design captured real-world screening preferences while maintaining scientific rigor and demonstrated strong preference for risk-based screening.

## INTRODUCTION

Despite advances in screening and treatment, over 40,000 US women still die of breast cancer annually. Mammographic screening remains the primary strategy for detecting breast cancer early, with the goal of reducing mortality. There have been no large mammographic screening trials since the 1980’s and 1990’s, despite the fact that our understanding of the biology of breast cancer has improved, as have our available treatment options.^1–6^ However, screening guidelines from US professional organizations differ substantially, as do system-wide screening programs across the world.^7^ While breast cancer screening is a critically important public health program, it falls short in that most clinically aggressive cancers still often present as palpable masses rather than screen-detected lesions; the increase in detection of precursor lesions (DCIS) has not been accompanied by a concomitant decrease in invasive cancers.^8^ There is clear room for improvement. Breast cancer is undoubtedly a complex and heterogeneous disease, and treatments are increasingly being tailored to tumor biology. One-size-fits-all treatment for breast cancer is no longer appropriate, so the question is whether a tailored approach can be extended to screening and prevention.

Scientific advances in germline profiling and radiomics provide the ability to define risk more accurately than ever before. This provides the opportunity to utilize each individual’s personal risk of breast cancer to inform screening frequency and modality, creating a modern, personalized alternative to current, unnuanced guidelines that consider the vast majority of women as ‘average’ risk.^9–11^

The WISDOM Study is a pragmatic, large-scale study comparing annual screening, the current standard of care in the USA, to a personalized approach in which women are provided personalized, risk-based screening recommendations commensurate with their individual breast cancer risk.^12^ It is powered for a primary endpoint of non-inferiority with respect to the number of late-stage cancers detected.^13^ WISDOM aims to establish whether screening based on a woman’s individual breast cancer risk is as safe and less morbid as the current US annual screening approach. Secondary endpoints aim to determine whether risk-based screening is acceptable and/or preferred by women, and if it can facilitate improved adoption of preventative interventions for high-risk women who are most likely to benefit.

The annual screening mammography arm was designed to reflect current standard of care in the United States; annual mammography starting at age 40 in accordance with American college of Radiology (ACR) guidelines.^14^ The personalized screening arm incorporated both clinical history and genetic predisposition (using the Breast Cancer Surveillance Consortium (BCSC) model,^15,16^ known breast cancer risk genes, and a polygenic risk model) into the WISDOM risk models to generate breast cancer screening assignment based on each participant’s individual risk.^17^

At the urging of WISDOM patient advocates, the study uses a preference-tolerant design that permits women who may be uncomfortable with randomization or who have strong inclinations towards one specific screening approach to participate in an adjunct observational cohort in which they self-select to receive annual or risk-based screening. Thus, the study enrolled two cohorts, referred to as the ‘randomized cohort’ and ‘observational cohort,’ each of which consists of two arms, Annual Screening and Risk-based Screening. The study is powered and the primary and secondary endpoints are analyzed exclusively using the population of women in the ‘randomized’ cohort.^13^

Here, we present a description of the baseline characteristics of the WISDOM Study population at the time of enrollment, including demographic, epidemiologic and breast cancer risk factors among each of the cohorts and arms. The design is detailed in the methods. The primary outcomes analysis will be presented later this year.

## RESULTS

### Participant recruitment and enrollment

**Figure 1:**
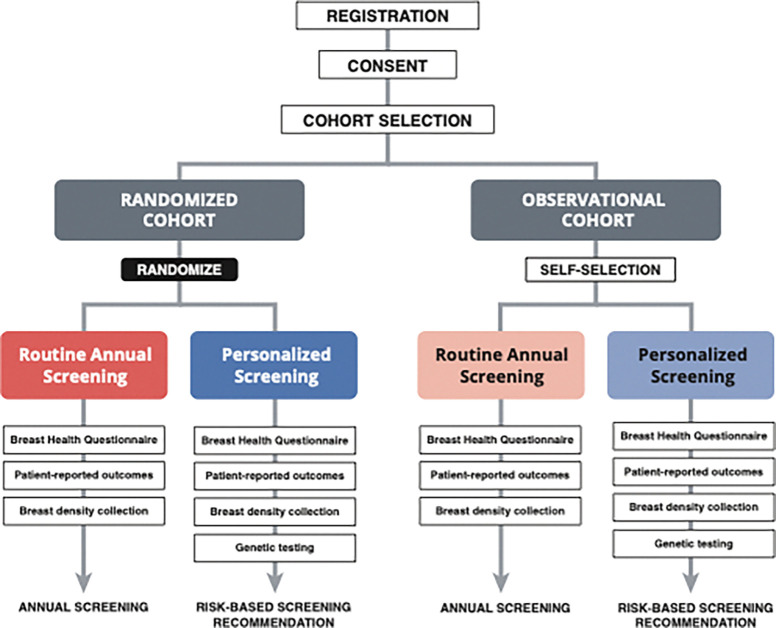
WISDOM Study’s preference tolerant design.

**Figure 2:**
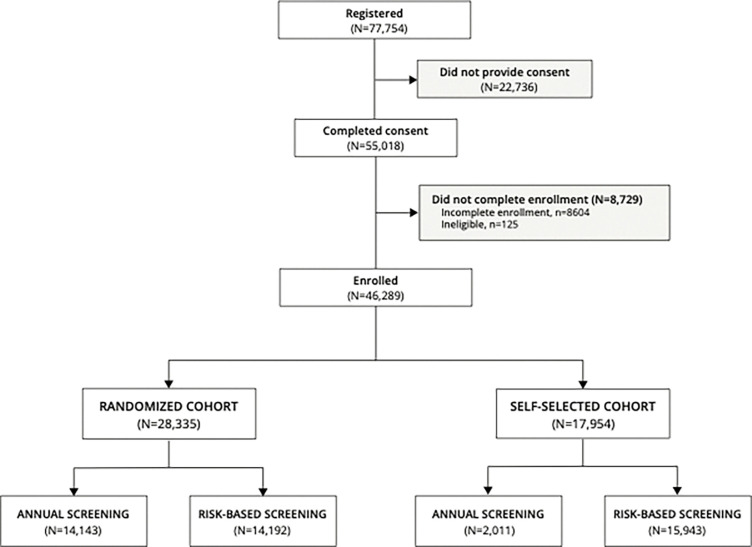
CONSORT Diagram

The WISDOM Study was open for enrollment for women between the ages of 40–74 who had not prior history of breast cancer, between August 31, 2016 and February 28, 2023. Interested women had the choice of enrolling in a randomized cohort or a parallel observational cohort in which they could self-select annual or risk-based screening (Fig. 1). A total of 77,754 women registered on the study website (wisdomstudy.org) by filling out basic eligibility and contact information (Fig. 2). Following registration, enrollment required additional steps, including informed consent, choosing which cohort they desired (randomized or observational), and if the latter, self-selection of risk-based or annual screening. Of those who registered, 22,736 did not complete informed consent.

In the online WISDOM enrollment workflow, consented individuals are required to complete a breast health questionnaire to assess baseline demographics, self-reported breast cancer risk factors and other epidemiological information. A total of 8,729 registered women were also excluded for failure to complete all enrollment steps (e.g. missing baseline questionnaires, n = 8,604) or ineligibility upon review of baseline questionnaires (age ineligibility, n = 124 and prior breast cancer diagnosis, n = 1). A total of 46,289 women completed consent and enrollment – 28,335 (61%) elected to be randomized, while 17,954 (39%) opted for self-selection. Among the latter, 15,943 (89%) self-selected risk-based screening.

### Demographic characteristics

In the randomized cohort, the mean age of participants was 54.8 years (s.d. 9.6), with no discernable difference in age between the annual and risk-based screening arms (Table 1). Annual and risk-based arms had similar racial and ethnic make-ups, with 77% self-identifying as non-Hispanic (NH) White, 9% Hispanic, 6% Black or African American and 4% Asian. The majority of the cohort (77%) was college educated. Similarly, there were few differences between those self-selecting annual versus risk-based screening, although it appears that older women tended to select annual screening more often, while younger women tended to self-select risk-based. Additionally, a larger number of NH White participants selected the annual arm (76%) over the risk-based arm (73%).

**Figure 3:**
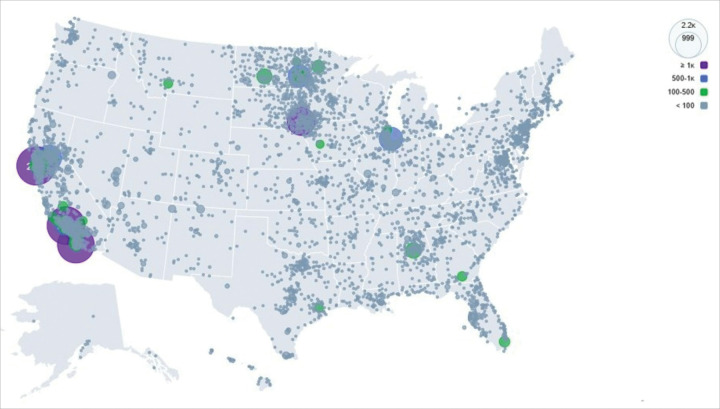
Geographic distribution of participants. Map illustrating the geographic density of enrolled participants in the WISDOM Study.

Overall racial and ethnic diversity improved with expansion to additional recruitment sites and Veteran’s Affairs (VA) outreach starting in 2020. This led to a greater proportion of people of color in the study, including an improvement from 1.7% Black or African American participation from 2016–2019 to 11.5% in 2022 (**Supplementary Table S1**). Geographically, although clinical sites focused recruitment efforts on local populations (see list of sites in **Supplementary Table S2**), women from across the US could enroll in WISDOM online. As such, the overall WISDOM population consists of women from every state in the country (Fig. 3), with highest concentrations of participants around recruitment sites. Most participants (54%) resided in the least deprived (Area Deprivation Index,^18^ or ADI of 1–3) areas and a minority lived in the most deprived (ADI 8–10) areas (18%).

### Epidemiologic Characteristics and Breast Cancer Risk Factors

In the randomized cohort, 12,856 of 28,335 participants (45%) reported no known first- or second-degree family history of breast cancer; 5703 (20%) reported a first-degree relative with breast cancer (Table 2). Prior breast biopsy was reported in 6333 (23%) participants in the randomized cohort, with atypia observed in 248 (1%) participants. Among all epidemiologic characteristics -- family history, previous biopsies reported age of menarche, age at first live birth, menopausal status, age at first live birth, menopausal status, use of menopausal hormone therapy (MHT), and use of risk-reducing medications – were similar between annual and risk-based arms in the randomized cohort. Epidemiologic characteristics of participants in the observational cohort were similar to those in the randomized cohort, with the exception of a higher proportion (29%, or 5,188 of 17,954) of women reporting a first-degree relative with breast cancer in the former.

Mammographic breast density was clinically validated for 85% of participants; 7% of participants across both cohorts had no prior mammogram before joining WISDOM and 83% of those screened previously had been screened in two years prior to joining the study. In both the randomized and observational cohorts, breast density was similar between cohorts and across arms, with 45% of participants assessed with dense breasts [heterogeneously dense (BIRADS C) or extremely dense (BIRADS D)], with only 8% with extremely dense breasts. Mean risk scores using the BCSC model^15,16^ were comparable across study arms, with annual arm mean 5-year BCSC score of 1.62 and risk-based arm 1.52. All risk model inputs are shown in Table 2 by study group and arm.

For those in the risk-based arm, completion of genetic testing was equal across the randomized and observational groups with 77% completion, of which 3–4% were found to have a pathogenic mutation. Detailed analyses of genetic findings are presented separately by pathogenic variant status and polygenic risk scores.^19^

### Participant differences according to self-selection vs. Randomization

Participant-level factors that contributed to the decision of whether to select randomization, self-select annual or self-select risk-based are shown in **Supplementary Table S3**. Differences were small, although older women were more likely to self-select into the annual arm than select randomization, whereas younger women tended to be more likely to self-select into the risk-based arm rather than elect to be randomized. Those who self-report as White NH were more likely to elect to be randomized, whereas Black or African American participants were more likely to self-select annual screening rather than randomization. Those who selected the risk-based arm were more likely to have a stronger family history and past atypia findings than those who chose to be randomized. Finally, the most disadvantaged participants were less likely to self-select risk-based screening and more likely to choose randomization.

## DISCUSSION

The WISDOM Study is a nationwide effort to prospectively compare personalized, risk-based breast cancer screening with standard-of-care annual mammography. We report several key findings upon completion of baseline enrollment. The first is that a personalized approach to screening based on individual breast cancer risk is broadly acceptable to women of screening age. The willingness of ~ 60% of the population to be randomized indicates that women are open to new approaches, including the risk-based approach. Importantly, in those who chose not to be randomized, 85% self-selected assignment to the risk-based arm, regardless of geography or year of enrollment. Risk-based screening therefore appears not only acceptable, but for many women, preferable.

Factors contributing to women’s willingness to be randomized may include educational recruitment materials that strongly encouraged randomization, a growing public awareness of debate surrounding potential harms of breast cancer screening,^20^ and confusion over multiple different recommendations available to each woman.^7^ Anecdotally, many women expressed an interest in knowing and better understanding their individual risk, contributing to better data upon which to make screening decisions, even ahead of the popularity of a more personalized approach to medicine that became more common after 2020.

Importantly, 77% of women completed genetic testing via the home testing kit provided by Color via the WISDOM Study, demonstrating that population-based genetic testing in a decentralized manner is both of interest to women and feasible in terms of implementation. We previously published data showing that genetic testing with post-result counseling is not associated with harm and can manage risk-related anxiety.^21,22^ As genetic testing is at the core of modern risk assessment, these results clearly indicate that implementation of population-based risk assessment, and indeed risk-based screening, is achievable and is of interest to the vast majority of women of screening age. The significance of this is underscored by the finding, published separately, that 30% of WISDOM participants who knew of no family history of breast cancer were found to have a pathogenic variant in one of nine breast cancer susceptibility genes (Fergus, K. et al, Submitted). This argues strongly that implementing population-based genetic testing of women between 25–30 would have an immediate and material impact in the prevention of some of the most aggressive and hard to treat breast cancers.

The enrollment of a diverse, representative population was a key goal of the WISDOM team. The study team continuously monitored WISDOM participant characteristics and noted that despite opening the trial across the United States, the trial participants were not initially reflective of the US population at large. As expansion to nationwide recruitment strategies were implemented (through additional grant support – PCORI, NCI RO1, BCRF), WISDOM’s racial/ethnic, geographic, and socioeconomic representation improved significantly. This is evident by the increase in Black or African American participation following intentional recruitment efforts by our expansion sites, dedicated Community Leadership Advisory Board, community engagement strategies, and most notably through our partnership with the VA.

The interest in personalized screening appears to span a large demographic swath. WISDOM Study participation spanned every state in the USA, and through targeted recruitment efforts, increasingly included a diverse population of women as the trial progressed, making it more reflective of the population of the United States. It also spanned both rural and urban populations. While the proportion of women with a college education dropped as the trial proceeded, it remains substantially higher than the US average (77% vs 47%). This likely reflects the recruitment approach that included outreach (usually by email) from many academic clinical centers, and the complex nature of the topic.

The preference-tolerant design enabled participation by women unwilling to be randomized, a substantial fraction of the population that would otherwise have been excluded. The randomized cohort will be used for the primary endpoint (the rate of stage 2B cancers), but there are a number of other questions where the self-selected cohort can be combined with the randomized group to better represent the population with increased power. Furthermore, this method helps shed light on biases resulting from including only individuals willing to be randomized in trials. By including all eligible participants interested in the trial, we can also examine the participant characteristics and preferences for those who chose to self-select their screening arm. In the self-selected group, we noted enrichment for women with classic risk factors for breast cancer compared with the general population including a family history of breast cancer or prior breast biopsy. While we found that participant characteristics such as age, race/ethnicity, and various breast cancer risk factors such as family history were associated with the decision to randomize or self-select an arm, these differences were small. It should be noted that the large size of the cohort is a strength of this study, however, this also means that small differences that aren’t clinically significant become statistically significant in comparing baseline characteristics, therefore p-values are not included in Table 1.

WISDOM’s pragmatic approach and virtual enrollment model also removed common trial participation barriers such as travel, time away from work, cost and other factors. Women were able to participate from any location they desired and at any time of day. The WISDOM team was available by email and phone to answer questions and assist with the study processes as needed. Although virtual trials are relatively new, internet-based trial approaches can come with several trade-offs, including over-representation of younger participants and additional effort required to promote protocol adherence and study retention compared to traditional trials.^24^ This age-dependent comfort with online activities may contribute to the WISDOM population’s skew towards women in the lower range of 40–74.

Among the limitations of the study are the slight enrichment for women with a family history of breast cancer, likely a natural result of such women’s heightened concerns regarding their own breast cancer risk. Similarly the WISDOM population is over-represented by women with college degrees compared to the general population. Although much effort was invested in raising awareness of the study in minority populations, the overall and randomized cohort are still marginally over-represented by non-Hispanic white women; Black or African American women, for example, who make up approximately 14% of American women in this age range comprise just 6% of the randomized population, which is similar to other trial representation.^25^

There is a similar study on risk-based screening in Europe (MyPEBs) being conducted in the setting of six countries with national screening programs.^26,27^ Work is underway to compare the populations and compare the results when results are reported in early 2027 (personal communications, Suzette Delaloge and Laura Esserman).

A personalized approach to screening that includes comprehensive genetic testing is feasible. The majority of women chose to be randomized and of these who chose their study arm, the vast majority chose the personalized approach. We are eagerly anticipating the outcomes which are under review and are expected to be reported at the end of 2025.

## METHODS

### Study Design

The WISDOM Study (NCT02620852) was a pragmatic randomized clinical trial comparing annual screening versus a novel risk-based approach. The primary endpoints were designed to test whether a personalized, risk-based approach to screening would be as safe, less morbid, acceptable to women and conducive to the uptake of preventive interventions in high risk women.^12,13,17^ The endpoints specifically were to evaluate: 1) non-inferior detection of late-stage cancers (stage IIB or higher) in the personalized screening arm; and 2) fewer biopsies as a measure of morbidity in the personalized screening arm by risk group. The secondary objectives included participant preference, endocrine risk reducing medication utilization rates, breast cancer worry and general anxiety.

The study was entirely virtual – no in-clinic visits were required. Consent, enrollment and participation in the study were largely conducted online through the study website, with data collection primarily through baseline and annual breast health and other questionnaires, along with access to participants’ electronic health records.

WISDOM used a preference-tolerant design encouraged by patient advocates that included an observational cohort for women who were not willing to be randomized, in which they could self-select whether to be part of the annual screening arm or the risk-based screening arm (Supplementary Figure S1). Therefore, each of the cohorts, randomized and observational, consisted of two arms each, the annual and risk-based screening arms. Per protocol, primary outcomes will be evaluated in the randomized arm only.

### Trial Oversight

The WISDOM Study was designed and implemented by a team of investigators from the Athena Breast Health Network, after consultation with a wide array of stakeholders that included patient advocates, relevant health professionals, industry representatives (from biotechnology, device, pharma and health insurance representatives), as well as representatives of the FDA and a number of charitable and professional medical societies. All participants provided written informed consent via electronic means. The WISDOM protocol was approved by the University of California San Francisco Institutional Review Board (approval #15–18234), which serves as the central IRB of record for all study sites. The study is overseen by a Data and Safety Monitoring Board (DSMB) that meets twice per year to review study progress, safety and outcomes. WISDOM complies with all local and national regulations regarding the use of human study participants and was conducted in accordance with the criteria set by the Declaration of Helsinki.

### Eligibility & Patient Recruitment

The WISDOM Study was open to females, aged 40 to 74 years old, who had no prior breast cancer or ductal carcinoma *in situ* (DCIS) diagnoses, were fluent in English or Spanish, and had no prior prophylactic bilateral mastectomy. Participants who were unable to provide consent or were not proficient in English or Spanish (as of June 2019) were excluded from the study.

Participants in the WISDOM Study were recruited via a number of outreach activities, initially through the Athena Breast Health Network^28^ sites that included University of California campuses (San Francisco, Davis, Los Angeles, San Diego and Irvine) and the Sanford Health system (North/South Dakota). Study enrollment was subsequently expanded in 2019–2020 with recruitment centers at the University of Chicago, University of Alabama-Birmingham, Diagnostic Center of Miami (Florida) and Louisiana State University (**Supplementary Table S2**). Additionally, WISDOM expanded enrollment to any eligible individuals across the US in 2019, and participants were assigned to the closest recruitment center for study coordination. In 2020, WISDOM partnered with Veterans Affairs (VA) to invite eligible veterans from select VA Health Care Systems through a passive recruitment approach. Recruitment methodology included direct patient emails and health portal messages, friend or family referrals, physician or medical team, community events and partnerships, mass media and social media, insurance outreach to members, and mailed study information, among others.

Interested participants used the WISDOM Study website to learn more about the study. For those without internet access, onsite enrollment was available at the recruitment sites by study coordinators. The study website provided detailed information including the rationale, lay descriptions of annual and risk-based screening, their potential harms and benefits, and ways of participating, along with descriptions of all study procedures. The goal was to provide sufficient information to facilitate virtual enrollment. Information was also provided concerning the rationale for performing a randomized clinical study, and the importance of participating in the randomized arm of the study. After education about the study on the website, participants registered and created an online study account to check eligibility, then were offered the opportunity to undergo the IRB-approved web-based informed consent process (Docusign, San Francisco, CA), in which enrollees selected whether they wanted to participate in the randomized or observational cohort. Electronic consent forms and information were offered in English and Spanish (as of 2019), and participants with questions were able to reach out to the study team via email and telephone. Consent included completion of a Release of Information form and HIPAA authorization to enable the study team to access personal electronic health records, per study protocol.

Enrollment was considered complete after informed consent was provided, baseline questionnaires were completed and a study arm assigned, whether via randomization or self-selection.

### Randomization

Consenting participants enrolling in the randomized cohort were randomized equally between personalized and annual screening arms. Randomization was stratified by recruitment site, age less than 50, mammogram on file (no prior mammogram, mammogram but not on file, mammogram on file), and BCSC risk score^15,16^ (3 categories) to ensure similar population distributions in the two arms. Once randomized, the assignment was kept for the duration of the trial with an *a priori* intention to treat analysis plan.

### Study Workflow

Following consent, participants completed a breast health questionnaire (BHQ) (components included in Table 1). Those in the personalized arm were sent saliva genetic testing kits to their home. If mammograms were done previously, reports were collected by study personnel or uploaded by participants to their study portal.

Once all risk information was gathered, participants were delivered screening assignment letters and encouraged to follow the recommendations from the study team. Those identified at elevated risk were offered a Breast Health Specialist (BHS) consultation and access to the Breast Health Decisions education tool.

Follow-up surveys were sent each year to ascertain changes to risk factors (e.g. new biopsies, additional family members diagnosed) and updated imaging results (e.g. density, biopsies) were collected in order to update screening assignments. Participants were given updated screening recommendation letters and those identified at elevated risk in follow-up years were offered a BHS consultation at the time of their high-risk assignment. Enrolled participants were followed longitudinally for at least 3 years (up to 9 years), and data on breast cancer incidence, unintended effects, patient acceptability and healthcare utilization were collected. The study team was blinded to outcomes data throughout the study, with only DSMB and lead study statistical team review of unblinded data.

Participants who indicated a diagnosis of DCIS or breast cancer through their questionnaire, phone, or email were contacted by coordinators to request additional information related to their diagnosis. This included obtaining pathology, oncology, surgical, and/or radiology reports to confirm the diagnosis information. Women who were diagnosed no longer received Screening Assignment Letters, but were provided a document of resources, and offered an opportunity to connect with our breast health advocates and participate in future studies.

### Baseline Surveys

In order to meet enrollment criteria in both cohorts, all participants were required to complete a breast health questionnaire (BHQ) that collected a range of demographic, epidemiologic and breast cancer risk information. A Patient-centered Outcomes (PCO) group was established to study and monitor breast cancer worry, perceived risk and anxiety. Participants completed a baseline PCO survey, with a second administered after the screening plan was received by the WISDOM participant.

### Yearly Surveys

All participants in both cohorts were asked annually to complete updated BHG and PCO questionnaires, along with requests to update any demographic information that may have changed. Survey requests were sent via email with links to online forms. For participants in the personalized screening arm, annual BHQ responses were used to update risk analysis and screening assignment each year. Participants were also instructed to contact the study team if any of their risk factors change (new family member diagnosed, new lump/bump found), if they require additional testing (biopsy or imaging), or received a diagnosis of DCIS or invasive breast cancer. Additionally, participants completed PCO surveys and reported endocrine risk reduction use through yearly questionnaires.

### Genomic Testing

Participants assigned to a risk-based screening arm were provided with a saliva collection kit for genetic analysis (Color Health, Burlingame, CA) that included both written and video instructions on sample collection and prepaid postage for return. Samples were processed by Color Health, who provided results provided to the study team within 4–10 weeks for review and incorporation into the personalized risk assessment. Leftover saliva specimens were returned to the study team for storage in the WISDOM Study central biorepository at Sanford Health.

### Imaging

Results from recent screening mammograms were obtained directly from the participant’s electronic medical record by study site, uploaded by the participant to their study portal, or by electronic fax. Date of screen, BIRADS (Breast Imaging-Reporting and Data System) classification, BIRADS density, and imaging modality (2D, 3D, or MRI) were recorded. If the participant reported a mammogram, but the clinical results were not obtained after 12 weeks of record retrieval attempts, a probability algorithm was used to impute their density for the purposes of randomization and screening assignment wherein those ages 40–49 were given BIRADS density C and those ages 50 + were given BIRADS density B (population averages by age). If the participant was aged 40–49 and reported no prior mammogram, their density was estimated to the highest risk category (BIRADS density category D) to determine the participant’s baseline risk. The study team iteratively retrieved actual density scores throughout the trial with the goal of at least one actual density score for each participant during the study.

### Risk Assessment

The breast cancer risk assessment model used in WISDOM has been previously described.^17^ The Breast Cancer Surveillance Consortium (BCSC) 5-year risk model that includes breast density as a risk factor,^15,16^ was calculated at baseline for all WISDOM participants and updated yearly based on questionnaires and updated breast density if available.

In cases where genomic analysis was not completed (not returned, inadequate sample, etc.) personalized screening assignments were based on the BCSC score only until such time as a test was received. If the participant returned a kit at a later time, their risk profile was updated and a new screening assignment generated to reflect her genetic risk contribution. As described by Fergus et al.,^19^ a panel of nine genes (BRCA1, BRCA2, ATM, CDH1, CHEK2, PALB2, PTEN, STK11 and TP53) was used to identify uncommon high- and moderate-penetrance mutations. A polygenic risk score (PRS) based on the presence of variant risk alleles derived from targeted sequencing was calculated using results of common single nucleotide polymorphisms (SNPs) known to increase breast cancer risk.^29,30^ These are described in Fergus et al. and included adjustment for self-reported racial and ethnic group.^19^

### Screening Assignments

The assignment of risk categories based on high and moderate penetrance mutations and polygenic risk has been previously described in detail^17,19^. **Supplementary Table S4** provides a summary of risk categories and related screening assignments used. For non-carriers, BCSC scores were combined with PRS in a Bayesian manner to calculate the 5-year risk of breast cancer. These results were then stratified into screening assignments. Updates to this risk assessment strategy and participant screening assignment were conducted at yearly intervals to accommodate the expansion of SNP lists, and changes in BCSC score variables reported by participants.

Participants in the annual arms were assessed using the BCSC 5-year risk model only. Participants in the highest 2.5% for their age group according to the BCSC risk model are flagged as elevated risk, given a Patient Risk Report and given the option to contact a Breast Health Specialist (BHS) provided by the trial to discuss their risk and suggest follow-up or referral for genetic counseling or high-risk clinic, if appropriate.

### Counseling for women identified as high risk using the Breast Health Decisions Tool

Pathogenic mutations are reported to participants via a WISDOM BHS and participants with these variants were considered elevated risk. Women detected as carriers of pathogenic variants received genetic counseling and risk reduction counseling from a WISDOM BHS and were offered more intensive screening and prevention options appropriate to their specific mutation and other variables. Variants of unknown significance were not reported to participants and did not inform screening assignments. Any change in variant classification to pathogenic throughout the trial triggered a patient notification and risk re-assessment.

In the Personalized Arm, participants at the highest 2.5% of risk by age were actively offered consultations from our Breast Health Specialist team as previously described^31,32^ to review their risk factors and tailored recommendations for enhanced screening and risk-reduction using the Breast Health Decisions (BHD) tool. Participants were provided with a BHS consultation summary, BHD tool summary report, encouraged to share their results with their local provider, as well as to connect with local genetics and/or high-risk breast centers for follow-up.

### Breast Health Decisions Tool

The Breast Health Decisions (BHD) Tool^33–35^ was offered to those in the Personalized arm without a pathogenic variant. The tool intended to educate women about their risk, put their risk in context compared to an average woman of her same age and race/ethnicity, and display the benefits of certain risk reducing strategies, such as prevention medications, lifestyle changes, and risk reducing procedures. The tool is populated with the participant’s responses from the study questionnaires, calculated risk information, and genetic information (polygenic risk). Follow-up surveys to assess the tool’s usability and self-reported actions of risk-reducing behaviors were offered as optional surveys.^32,36^ At the end of the tool, the participant could access and download a summary of the tool from her WISDOM Study portal and was encouraged to share their results with their local provider.

### Risk Assignment Review

We formed a Screening Review Board (SRB) in 2018 to incorporate more clinical judgement for participants who had complex risk factors or were close to the risk threshold cut-points. The SRB was comprised of at least 1 clinician (primary care provider with expertise in either genetics or breast cancer risk assessment), 1 genetics lead (Cancer Genetics Clinic Directors), 1 Breast Health Specialist (genetic counselor and/or PhD/MD level trained breast health expert), and study staff. The group met at least once per month to review clinical and risk output variables to determine if the assigned screening frequency met criteria to shift the assignment to a more frequent screening bucket. Participants who were recommended to change assignments were reviewed by the study Principal Investigator in conjunction with the Risk Thresholds Working Group to make a final determination of risk assignment. These reviews informed adjustments to risk assignments over the course of the trial.

### Statistical Analysis

The study was powered for comparison of the rate of stage IIB or higher cancers between women randomized to an annual screening recommendation versus those randomized to a risk-based screening recommendation with a non-inferiority margin of 0.05% per year, assuming annual incidence rates of 95 cancers per 100,000 women in each arm. Baseline characteristics of the study cohort were summarized using descriptive statistics appropriate to each variable type. Marginal differences across study groups and screening arms were evaluated using standard statistical tests (e.g., chi-square tests). To account for interdependencies among characteristics, we included variables shown to have statistically significant relationships or large effect sizes in simple bivariate analyses and used a multivariable generalized linear model with a log link to assess differences not explained by associations with other characteristics. Complete cases account for approximately 72% of total sample and we used multiple imputation to account for missing values. Comparison of results for complete cases and total sample after imputation show similar results. All statistical analyses were conducted using R (version 4.4.1; R Foundation for Statistical Computing, Vienna, Austria).

## Supplementary Material

Supplementary Figure

Supplementary Figure S1 is not available with this version.

Supplementary Files

This is a list of supplementary fi les associated with this preprint. Click to download.

• WISDOM.BaselineSupplementFINAL.pdf

## Figures and Tables

**Table 1:** WISDOM Cohort Demographic Characteristics

	Randomized		Observational	
	Annual (N = 14,143)	Risk-based (N = 14,192)	Annual (N = 2,011)	Risk-Based (N = 15,943)
**Age**, n (%)				
40–49	5203 (37%)	5209 (37%)	523 (26%)	6654 (42%)
50–59	4310 (30%)	4412 (31%)	603 (30%)	4805 (30%)
60–69	3691 (26%)	3658 (26%)	654 (33%)	3678 (23%)
70–74	939 (7%)	913 (6%)	231 (11%)	806 (5%)
**Race, Ethnicity**, n (%)				
NH White	10884 (77%)	10920 (77%)	1529 (76%)	11673 (73%)
Hispanic	1237 (9%)	1313 (9%)	177 (9%)	1715 (11%)
NH Asian	584 (4%)	560 (4%)	106 (5%)	888 (6%)
NH Black or African American	808 (6%)	809 (6%)	132 (7%)	841 (5%)
NH More than one race	431 (3%)	418 (3%)	36 (2%)	558 (3%)
NH Native Hawaiian or Other Pacific Islander/American Indian or Alaska Native	69 (0.5%)	68 (0.5%)	6 (0.3%)	59 (0.4%)
Other/Unknown	130 (1%)	104 (1%)	25 (1%)	209 (1%)
**Educational attainment**, n (%)				
High school graduate or less	460 (3%)	477 (3%)	70 (3%)	460 (3%)
Some college or technical school	2861 (20%)	2804 (20%)	375 (19%)	3082 (19%)
College graduate or more	10789 (76%)	10869 (77%)	1555 (77%)	12364 (78%)
No Response/Missing	33 (0%)	42 (0%)	11 (1%)	37 (0.2%)
**ADI**, n (%)				
Least Disadvantaged	7523 (53%)	7510 (53%)	1117 (56%)	8748 (55%)
Middle Disadvantaged	3485 (25%)	3561 (25%)	474 (24%)	3925 (25%)
Most Disadvantaged	2609 (18%)	2660 (19%)	336 (17%)	2630 (16%)
Data suppressed/Could not be geocoded	526 (4%)	461 (3%)	84 (4%)	640 (4%)

**Table 2:** Epidemiologic and Breast Cancer Risk Factors at Study Entry

	Randomized		Observational	
	Annual (N = 14,143)	Risk-based (N = 14,192)	Annual (N = 2,011)	Risk-Based (N = 15,943)
**Family History, n (%)**
None^†^	6447 (46%)	6409 (45%)	948 (47%)	5448 (34%)
1° Relative	1696 (12%)	1724 (12%)	272 (14%)	2657 (17%)
2° Relative	3400 (24%)	3438 (24%)	452 (22%)	4057 (25%)
1°and 2° Relative	1123 (8%)	1160 (8%)	170 (8%)	2089 (13%)
Don’t Know	1263 (9%)	1268 (9%)	141 (7%)	1445 (9%)
No Response/Missing	214 (2%%	193 (1%)	28 (1%)	247 (2%)
**Age (yrs) at Menarche**, n (%)				
Under age 12	2,425 (17%)	2,472 (17%)	290 (14%)	2,628 (16%)
12 to 13	6,485 (46%)	6,392 (45%)	871 (43%)	7,227 (45%)
14 or above	2,854 (20%)	2,860 (20%)	393 (20%)	3,198 (20%)
Unknown/Missing	2,379 (17%)	2,468 (17%)	457 (23%)	2,890 (18%)
**Age (yrs) at First Birth**, n (%)				
Nulliparous	2,958 (21%)	2,870 (20%)	394 (20%)	3,350 (21%)
< 20 years	964 (7%)	1,031 (7%)	117 (6%)	993 (6%)
20–24 years	2,144 (15%)	2,120 (15%)	282 (14%)	2,109 (13%)
25–29 years	2,459 (17%)	2,454 (17%)	336 (17%)	2,581 (16%)
30–34 years	1,919 (14%)	1,896 (13%)	250 (12%)	2,322 (15%)
> 34 years	1,233 (9%)	1,302 (9%)	162 (8%)	1,631 (10%)
Unknown/Missing*	2,466 (17%)	2,519 (18%)	470 (23%)	2,957 (19%)
**Prior Biopsy at Baseline**, n (%)				
Not Biopsied	11,025 (78%)	10,953 (77%)	1,465 (73%)	11,739 (74%)
Biopsied	2,986 (21%)	3,099 (22%)	520 (27%)	3,883 (24%)
Biopsied - ADH	119 (1%)	129 (1%)	25 (1%)	292 (2%)
Unknown/Missing	13 (< 1%)	11 (< 1%)	1 (< 1%)	29 (< 1%)
**Menopausal status at Baseline**, n (%)				
Pre-Menopause	4,118 (29%)	4,107 (29%)	436 (22%)	5,281 (33%)
Post-Menopause	7,708 (55%)	7,694 (54%)	1,130 (56%)	7,861 (49%)
Unknown/Missing	2,317 (16%)	2,391 (17%)	445 (22%)	2,801 (18%)
**Age at Menopause at Baseline**, n (%)				
Pre-Menopause	4,118 (29%)	4,107 (29%)	436 (22%)	5,281 (33%)
< 40 years	1,224 (9%)	1,255 (9%)	142 (7%)	1,346 (8%)
40–44 years	973 (7%)	1,009 (7%)	149 (7%)	1,058 (7%)
45–55 years	4,794 (34%)	4,770 (34%)	745 (37%)	4,768 (30%)
> 55 years	570 (4%)	509 (4%)	71 (4%)	502 (3%)
Unknown/Missing	2,464 (17%)	2,542 (18%)	468 (23%)	2,988 (19%)
**Hormone Replacement at Baseline**, n (%)				
None	7,919 (56%)	7,858 (55%)	942 (47%)	8,640 (54%)
One or More	3,889 (27%)	3,924 (28%)	622 (31%)	4,465 (28%)
Unknown/Missing	2,335 (17%)	2,410 (17%)	447 (22%)	2,838 (18%)
**Chemoprevention at Baseline**, n (%)				
None	10,394 (73%)	10,403 (73%)	1,264 (63%)	11,685 (73%)
One or More	161 (1%)	183 (1%)	27 (1%)	269 (2%)
Unknown/Missing	3,588 (25%)	3,606 (25%)	720 (36%)	3,989 (25%)
**Mammographic breast density** ^‡^				
Almost entirely fatty (A)	686 (5%)	723 (5%)	136 (7%)	677 (4%)
Scattered fibroglandular densities (B)	4,997 (35%)	5,242 (37%)	787 (39%)	5,177 (32%)
Heterogeneously dense (C)	4,945 (35%)	5,151 (36%)	826 (41%)	6,341 (40%)
Extremely dense (D)	1,039 (7%)	1,002 (7%)	158 (8%)	1,434 (9%)
No Response/Missing	2,476 (18%)	2,074 (15%)	104 (5%)	2,314 (15%)
**Last mammogram prior to WISDOM**				
Never had mammogram	894 (6%)	896 (6%)	60 (3%)	1190 (7%)
< 2 yrs	11932 (84%)	12001 (85%)	1866 (93%)	12904 (81%)
2–3 yrs ago	736 (5%)	717 (5%)	39 (2%)	1034 (6%)
≥ 4 year/Stopped mammograms	447 (3%)	447 (3%)	27 (1%)	670 (4%)
Don’t Know/NR/Missing	134 (1%)	131 (1%)	19 (1%)	145 (1%)
**Breast cancer genetic test**				
Tested	N/A	10,877 (77%)	N/A	12,221 (77%)
Not Tested/Missing Result	N/A	3,315 (23%)	N/A	3,722 (23%)
**Pathogenic Mutation Status (Among Gene Panel Tested)**				
No Pathogenic Mutation Found	N/A	10,595 (97%)	N/A	11,789 (96%)
Pathogenic Mutation Found	N/A	282 (3%)	N/A	432 (4%)
High - BRCA1, BRCA2, TP53, PTEN, STK11, CDH1, PALB2	N/A	79 (28%)	N/A	155 (36%)
Moderate: ATM, CHEK2	N/A	203 (72%)	N/A	277 (64%)
**BCSC 5-Year Risk Score**				
Mean (SD)	1.60 (0.97)	1.50 (0.91)	1.71 (0.99)	1.58 (1.02)
Median [Min, Max]	1.41 [0.15,20.02]	1.30 [0.13,14.61]	1.50 [0.14,11.32]	1.35 [0.13,17.92]
No Response/Missing	34 (0.2%)	110 (1%)	0 (0%)	64 (0.4%)
**BCSC 5-Year Polygenic Risk Score**				
Mean (SD)	N/A	1.57 (1.38)	N/A	1.67 (1.55)
Median [Min, Max]	N/A	1.18 [0.05,21.55]	N/A	1.23 [0.04,35.10]
No Response/Missing, n (%)	N/A	252 (2%)	N/A	282 (2%)

* Unknown = No Response/Missing/Biologically Implausible.

† No first or second degree relative.

‡ Letters in parentheses refer to BIRADS density classifications

## Data Availability

Data were generated by the authors but are not publicly available while trial follow-up remains underway. Upon completion of the trial and reporting of the primary outcomes, we plan to provide summary statistics upon request to the study investigators, under terms set forth by the study’s institutional review board. Genetic data and test results are protected and will not be provided on a participant level. We are committed to collaboration as a large multi-site trial and welcome future collaborators to work with WISDOM 2.0, which is now enrolling. Please contact the corresponding authors for further information on collaborating with the WISDOM team.

## CODE AVAILABILITY

Code for the Breast Cancer Surveillance Consortium five-year breast cancer risk model is available online at https://www.bcsc-research.org/index.php/tools
